# Investigating genomic, proteomic, and post-transcriptional regulation profiles in colorectal cancer: a comparative study between primary tumors and associated metastases

**DOI:** 10.1186/s12935-023-03020-7

**Published:** 2023-09-05

**Authors:** Hersh Ham-Karim, Ola Negm, Narmeen Ahmad, Mohammad Ilyas

**Affiliations:** 1 Department of Pharmacy, College of Medicine, Komar University of Science and Technology, Chaq-Chaq-Qualaraisi, Sulaimani, Iraq; 2 Division of Medical Sciences and Graduate Entry Medicine, Faculty of Medicine and Health Sciences, School of Medicine, University of Nottingham, Nottingham, UK; 3 Kurdistan Institution for Strategic Studies and Scientific Research, Qirga, Sulaimani, KRG Iraq; 4 Nottingham Molecular Pathology Node, University of Nottingham, Nottingham, UK

**Keywords:** Primary tumour and corresponding metastasis, Gene mutations, NGS, miRNA, Protein, RPPA

## Abstract

**Introduction:**

Approximately 50% of patients with primary colorectal carcinoma develop liver metastases. This study investigates the possible molecular discrepancies between primary colorectal cancer (pCRC) and their respective metastases.

**Methods:**

A total of 22 pairs of pCRC and metastases were tested. Mutation profiling of 26 cancer-associated genes was undertaken in 22/22primary-metastasis tumour pairs using next-generation sequencing, whilst the expression of a panel of six microRNAs (miRNAs) was investigated using qPCRin 21/22 pairs and 22 protein biomarkers was tested using Reverse Phase Protein Array (RPPA)in 20/22 patients’ tumour pairs.

**Results:**

Among the primary and metastatic tumours the mutation rates for the individual genes are as follows:*TP53* (86%), *APC* (44%), *KRAS* (36%), *PIK3CA* (9%), *SMAD4* (9%), *NRAS* (9%) and 4% for *FBXW7, BRAF, GNAS and CDH1.* The primary-metastasis tumour mutation status was identical in 54/60 (90%) loci. However, there was discordance in heterogeneity status in 40/58 genetic loci (z-score = 6.246, difference = 0.3793, P < 0.0001). Furthermore, there was loss of concordance in miRNA expression status between primary and metastatic tumours, and 57.14–80.95% of the primary-metastases tumour pairs showed altered primary-metastasis relative expression in all the miRNAs tested. Moreover, 16 of 20 (80%) tumour pairs showed alteration in at least 3 of 6 (50%) of the protein biomarker pathways analysed.

**Conclusion:**

The molecular alterations of primary colorectal tumours differ significantly from those of their matched metastases. These differences have profound implications for patients’ prognoses and response to therapy.

**Supplementary Information:**

The online version contains supplementary material available at 10.1186/s12935-023-03020-7.

## Introduction

Although significant advances in adjuvant chemotherapy for colorectal cancer (CRC) were achieved in the past few decades, the 5 year survival rate for the disease is still poor and as such, more than half of all colorectal carcinoma patients are expected to die from metastasis within this period [1]. Thus the understanding of the biological mechanisms of metastasis will enable the institution of new diagnostic and therapeutic strategies to detect the disease in its early stages and consequently hinder its progression to the metastatic state [2]**.** Though metastases are the main cause of colorectal cancer deaths, much more is known about the underlying molecular features of the primary disease than the more advanced disease stages. This is because only a relatively limited number of studies have performed on metastatic colorectal carcinomas [3]. Overwhelming clinical evidence indicates that colorectal tumours which show identical histological features have dramatically different prognosis and response to treatment. Interestingly, the phenomenon of diverse clinical outcome supports the notion that colorectal cancer is a heterogeneous disease with molecular ambiguities [4, 5]. Moreover, the initiation and progression of tumours is unique among the individual patients [6]. As a consequence, researchers now focus on the molecular basis of this malignancy in order to explain the susceptibility, growth, progression, response to treatment and metastatic spread of the disease[7]. In general, the mutations which lead to primary cancers are also present in metastases, but additional alterations can occur during progression from primary to metastatic disease [8].

Generally, multiple and consecutive genetic alterations are needed for cancer development and some patients may have coexistent alterations in two or more different signalling pathways [9, 10]. CRCs accumulate mutations via the process of Darwinian evolution followed by clonal expansion and this can create spatial genetic heterogeneity among tumour cells. As cancer cells metastasize, different molecular alterations are required—and accumulated—to adapt to the new environments. Moreover, an older clone may spread to a distant site whilst the primary tumour progresses and metastasizes to some other sites, thereby creating more complex heterogeneity among primary and its various metastatic clones—temporal heterogeneity. The heterogeneity between primary and metastatic cancers is a leading cause of increased resistance to therapy, which is the major cause of cancer-related death [8, 11]. A better understanding of the molecular discrepancies between the primary tumours and their corresponding metastases is required for developing efficient targeted therapy to hinder the progression to metastatic disease and consequently improve patients’ survival.

Additionally, the characterization of oncogenic mutations in metastatic disease could represent potential therapeutic targets. However, obtaining biopsies from metastatic sites for molecular testing involves an invasive procedure. Therefore, patients and clinicians still prefer using the least invasive method possible for genetic testing. Recently, there has been a growing evidence suggesting that there exists inter- and intra-tumour genetic heterogeneity in a range of solid tumours including CRC, thereby raising the concerns that molecular profiling of primary tumours may not be representative of metastatic disease [12, 13]. Comparative sequencing studies of CRC found a high degree of concordance in somatic mutation profiles between primary CRC tumours and their matched metastases in a number of studies [14, 15]. In direct contrast, a study using next generation sequencing reported a high degree of discordance in mutation status between primary and metastatic samples of 21 patients [16].

In this study, we investigated the molecular discrepancies between primary colorectal cancer (pCRC) samples and their matched metastases to assess the mutation profiles of 22 primary-metastasis CRC pairs in 26 cancer-associated genes using Next-generation sequencing (NGS) [17]. Furthermore, the miRNA expression profiles of the tumour pairs were compared using RT-qPCR. Additionally, the expression of 22 well-characterized protein biomarkers of growth signalling pathways was evaluated by reverse phase protein array (RPPA) in the primaries and their matched metastases.

## Patients and methods

### Inclusion criteria

For this study, 22 pairs of pCRC and metastasis were selected. All patients had undergone surgery between 2004 and 2005 at the Queen`s Medical Centre (QMC) Nottingham, UK. Moreover, the candidate tumour blocks were selected based on the availability of clinicopathological data. Tumour cellularity, i.e. the proportion of tissue sections with cancer cells, was determined in the primary and metastatic CRC samples by a trained pathologist. The clinicopathological features of the cases included in this study is shown in Additional file 2: Data_General. Tumour cellularity data was available for only 21/22 tumour pairs. Samples were provided by the Nottingham Health Science Biobank and the study was approved by Nottingham Research Ethics Committee Reference number (REC reference C02.310). Moreover, all tissue samples obtained for this study are from adenocarcinoma origin and were collected via surgical procedures. Importantly, both the primary and metastatic samples were obtained simultaneously at the same timepoint.

### Molecular analysis

We previously reviewed the haematoxylin and eosin stained slides and performed DNA extraction [18]. Total RNA and miRNA isolation was performed using the miRNAeasy FFPE kit (Qiagen, Hilden, Germany).

#### Next generation sequencing (NGS) library preparation

Trusight™ tumour kit (Illumina, USA) which provides a comprehensive view of somatic variation in 82 exons of 26 genes involved in solid tumours and total length capture of 21.6 Kb. Genomic DNA from CRCs (primary and metastasis) FFPE was fragmented to 300 -330 bp segments. The primers, adapters and indexes were then ligated to the DNA fragments to construct libraries. The DNA fragments were hybridized and after enrichment, transferred into the template position in the MiSeq reagent cartridge and sequenced on the Illumina MiSeq sequencing platform using MiSeq reagent kits v2 as recommended by Illumina. The detailed procedure is in the Additional file 8: Data_NGS.

In order to validate the mutations detected by NGS, the samples were also analysed using the quick multiplex consensus-PCR (QMC-PCR) in conjunction with a high resolution melting (HRM) protocol as previously described [19].

#### miRNA quantification by real-time quantitative RT-PCR

After creating cDNA using miScript II RT kit (Qiagen, Hilden, Germany), the selected miRNAs were quantified using a miScript SYBR Green PCR kit (Qiagen) in a 7500 Fast Real-Time PCR System (Applied Biosystems). The miRNA-specific primer sequences and primer efficiencies obtained from assay optimization are listed in Additional file 3: miRNA. The ΔΔCt method of relative mRNA quantification was modified to quantify the relative miRNA expression between normal tissue and primary CRC, and between normal tissue and metastatic tumours using RNU6B as a reference gene [20, 21], as follows: step 1: ΔCt = (Ct target miRNA – Ct of RNU6B) and step 2: ΔΔCt = (ΔCt target miRNA primary tumour tissue—ΔCt target miRNA normal tissue; and ΔCt target miRNA metastatic tumour tissue – ΔCt target miRNA normal tissue). The obtained ΔΔCt was normalised to 100% tumour cellularity by using log2 fold change, where fold change = 100/TC, and TC = tumour cellularity expressed in percentage (see Additional file 3: Data_miRNA). The relative miRNA fold changes of tumour tissues were calculated by the Equation 2^−ΔΔct^, using the tumour cellularity-normalised ΔΔCt. Relative fold change of ≥ 2 (i.e. 0.5 ≤ relative ratios ≥ 2.0) between primary/metastatic tumours and normal tissue wastaken as the cut-off.

The TC-normalisation method is based on two assumptions: (i) any difference in ΔΔCt between tumour and normal tissues is caused by the proportion of the tumour sample containing tumour cells; (ii) all tumour cells are expressing the miRNA at the same levels.

#### Reverse phase protein assay (RPPA)

For protein extraction, 20 μm thick sections were macro-dissected from formalin-fixed paraffin-embedded (FFPE) tumour blocks to enrich for the tumour content. Then protein lysates were quantified and an equal amount of lysates were loaded into a 384-well plate and visualised using the 700 nm (red) channel on an Odyssey high-resolution scanner (LI-COR, Lincoln, USA) at 21 μm resolution. For comparative analysis, the intensity signals from total protein was used to normalize signals generated from other biomarkers [22]. Complete RPPA and tumour cellularity data were available for only 20 of the CRC tumour pairs.

### Statistical and data analysis

All calculations, unless otherwise stated, were performed in Excel spreadsheet version 2010.The Benjamini and Hochberg correction was applied to multiple testing at a false discovery rate of 0.05 (5%) using the online FDR calculator software (www.sdmproject.com/utilities/?show=FDR). *P* values and adjusted *P* values (for multiple testing) of < 0.05 were considered to be statistically significant.

#### Somatic mutations

Using the formula mutant allele frequency (MAF) X 2/ tumour cellularity, the heterogeneity scores (HS) were determined for individual mutations found in primary and metastatic CRC as previously described [23–25]. Then the HS of matched primary and metastatic CRC were compared for any correlation using the using Pearson’s correlation test in the online statistics software at http://vassarstats.net/index.html. Furthermore, the HS were used to categorize the somatic mutations as (i) present in a subpopulation of tumour cells (STC, HS < (0.95)), (ii) present in all tumour cells (ATC, HS = (0.95 to 1.05)), and (iii) present in a background of copy number variation (CNV, HS > (1.05)) [23–25]. Also, in some tumour pairs, there were corresponding wild type (WT, HS = 0) alleles for the mutant partners. Alleles which retained their background statuses (STC, ATC, CNV, or WT) in primary and metastatic pairswere further characterized to determine if there was an intra-status *change* in the background heterogeneity state in the metastatic tumour counterparts: any HS difference of ≥ 0.5 between the tumour pairs was regarded as significant change (the HS would change by ≥ 0.5 if 100% of the tumour cell population acquire or lose the mutant allele by amplification or deletion) (see Additional file 5: Data_Somatic Mutation Profile). Then, the proportion of total number of alleles which showed a *change* in the background heterogeneity status was calculated for the entire sample set using the Test for One Proportion (z-statistics) module in the MEDCALC easy-to-use statistical software at https://www.medcalc.org/calc/test_one_proportion.php. Differences in proportions were considered significant at a *P* value of < 0.05.

In addition, the mutant allele frequency ratios (MAFRs) were calculated relative to trunk and branch driver mutations as previously described [26]. Then, the Pearson’s correlation test was used to determine the level of correlation between primary and metastatic tumour MAFRs.

#### MicroRNA expression

Tumour cellularity-normalised microRNA expression levels in the primary and metastatic tumours were categorized into high, normal and low on the basis of their relative expression ratios, i.e. relative ratios (RR) ≥ 2 = high expression, RR between 0.5 and 2 = normal expression, and RR ≤ 0.5 = low expression. Then the concordance in miRNA expression between primary and metastatic tumours was sought using QuickCalcs Kappa calculator at (https://www.graphpad.com/quickcalcs/kappa2/).

Furthermore, the miRNA expression of primary relative to metastatic CRC (PM-RE) was determined using the ratio of the relative expression of primary tumour to that of the metastatic tumour. The cut-off for miRNA PM-RE change (up-regulation and down-regulation) was ≥ 2 (i.e. ≤ 0.5 primary-metastases ratio ≥ 2) (see Additional file 3: miRNA). The Test for One Proportion module in the MEDCALC easy-to-use statistical software was used to determine the statistical significance of the difference in proportions of altered and unaltered PM-RE for each of the miRNA biomarker. Differences in proportions were considered significant at a *P* value of < 0.05. Overall miRNA expression was regarded as discordant for the two tumour groups when ≥ 50% of the primary-metastasis pairs showed ≥ twofold change in PM-RE in ≥ 50% of the miRNA tested.

#### Protein expression

The relative expression of each protein biomarker was obtained for each sample by normalising the intensity signals of each biomarker to that of the total protein concentration [22]. The relative biomarker expression was further normalised to tumour cellularity using the formula: E^m^ + ((∆TC/100) x E^m^), where E^m^ = relative protein biomarker expression in metastatic tumour, ∆TC = difference in tumour cellularity between primary and metastatic tumour, expressed in percentage. The relative expression of each biomarker in the metastatic tumour was considered to be significantly altered (up-regulated or down-regulated) if the difference in the relative biomarker expression between the primary and TC-normalised metastatic tumour values (differential biomarker expression) was greater than the standard deviation of the biomarker expression for the entire tumour population, primary and metastatic tumours combined (Additional file 4: Data_RPPA Markers). We tested the validity of this approach by comparing the alteration status obtained for the protein biomarkers with that of their isoforms/modified forms (e.g. PTEN versus phosphoPTEN, phosphoAKT-serine versus phosphoAKT-threonine) and between proteins that are closely related functionally (e.g. CD34 versus CD31, RAS versus RASA1) for concordance.

To analyse the data in a biological context, all the protein biomarkers were categorized into the following seven (7) biological pathways using the KEGG pathway analyses at https://www.genome.jp/kegg-bin/show_pathway [27]: cellular adhesion molecules-epithelial mesenchymal transition pathway (CAM-EMT: CD34, CD31, D2-40, BEREP4, AE1-3 and E-cadherin), RAS-RAF-MEK pathway (RAS, RASA1 and pCRAF), PI3K-AKT-PTEN pathway (P85, P110, PTEN, phosphoPTEN, phosphoAKT-serine, phosphoAKT-threonine, mtor, and pGSK), TGFB-SMAD4 pathway (TGFB and SMAD4), apoptosis (BCL2) and a miscellaneous group, the transcriptional mis-regulation pathway (WT1 and KLF4). A pathway was regarded as altered for individual tumour pairs if ≥ 50% of *biomarkers* of that pathway was altered. This criterion took into consideration the functional redundancy of protein markers and their isoforms/modified forms. For example, the pairs of P85 and P110, PTEN and phosphoPTEN, and phosphoAKT-serine and phosphoAKT-threonine are the isoforms/modified forms of PI3K, PTEN and AKT respectively, and alterations in any of the forms was counted only once as an alteration in the parent biomarker for the tumour pairs. A sample pair was regarded as having altered expression profile if 50% or more of its analysed *pathways* was significantly altered. Furthermore, the biomarker expression pattern of the primary tumour group was considered significantly different from that of the metastatic tumours if ≥ 50% of the *tumour pairs* has altered expression profiles (Additional file 1: Data_Biomarker Pathways).

## Results

### Mutation profile

Somatic mutation profiling data was generated for all 22 tumour pairs. A total of 60 somatic non-synonymous mutations were found in 10 genes, comprising 51 specific types of mutations and 9 of which are recurrent. Of the 51 specific mutation types 15 are small indels, whilst 36 are single nucleotide variations (point mutations). All 9 recurrent mutation types are point mutations. Thirty-five of the single nucleotide mutations have previously been described, whilst one is a novel mutation (APC c.3871C > T). The 15 small indels are comprised of 9 deletions and 6 insertion (including 2 duplications). All indels, made up of 6 known and 9 novel mutations, generated a frame-shift (as shown in Table 1).

**Table 1:** Somatic Mutation Profile of paired Primary and Metastatic CRC

Case no.	Gene	Mutation	Codon	Exon	State	SIFT prediction	PolyPhen Prediction	FATHMM Prediction	Pri	Met
1	APC	c.3871C > T	1291	15	Novel	N/A	N/A	N/A	+	+
		c.4468_4474delCATTTTG	1490–92	15	Novel	N/A	N/A	N/A	+	+
	KRAS	c.35G > T	12	2		Deleterious (0)	Probably damaging(0.984)	Pathogenic (0.98)	+	Wild
		c.183A > T	61	3		Deleterious (0)	Possibly damaging(0.486)	Pathogenic (0.93)	+	+
	TP53	c.518T > C	173	5		Deleterious (0)	Probably damaging(0.996)	Pathogenic (0.99)	+	+
		c.536A > G	179	5		Deleterious (0)	Probably damaging(0.993)	Pathogenic (0.99)	+	Wild
	SMAD4	c.1496G > A	499	9		Deleterious (0)	Probably damaging(0.997)	Pathogenic (0.98)	+	+
2	APC	c.3707_3708delCA	1236	15		N/A	N/A	N/A	+	+
		c.4033G > T	1345	15		N/A	N/A	Pathogenic (0.85)	+	+
		c.4348C > T	1450	15		N/A	N/A	Pathogenic (0.90)	+	+
	KRAS	c.38G > A	13	2		Deleterious (0.03)	Possibly damaging(0.517)	Pathogenic (0.98)	+	+
	SMAD4	c.1478A > G	493	9		Deleterious (0)	Probably damaging(0.96)	Pathogenic (1.0)	+	+
	GNAS	c.2531G > A	844	8		Deleterious (0)	Probably damaging(1)	Pathogenic (0.99)	+	Wild
3	APC	c.4012C > T	1338	15		N/A	N/A	Pathogenic (0.90)	+	+
	NRAS	c.181C > A	61	3		Deleterious (0.01)	Possibly damaging(0.751)	Pathogenic (0.99)	Wild	+
	TP53	c.797G > A	266	7		Deleterious (0)	Probably damaging(0.998)	Pathogenic (1.0)	+	+
		c.761T > A	254	7		Deleterious (0)	Probably damaging(1)	Pathogenic (0.99)	Wild	+
4	KRAS	c.139G > C	47	2		Deleterious (0)	Probably damaging(0.986)	N/A	+	+
	TP53	c.517G > A	173	5		Deleterious (0.03)	Probably damaging(0.954)	Pathogenic (0.99)	+	+
	CDH1	c.1024A > T	342	8		Deleterious (0.02)	Benign (0.089)	N/A		Wild
5	BRAF	c.1799T > A	600	15		Deleterious (0)	Probably damaging(0.999)	Pathogenic (0.99)	+	+
	TP53	c.723delC	241	7		Tolerated (0.56)	Benign (0.033)	Neutral (0.36)	+	+
6	APC	c.3944C > A	1315	15		Damaging due to Stop	N/A	Pathogenic (0.78)	+	+
	TP53	c.54_55insAT	19	2	Novel	Deleterious (0)	Probably damaging(0.985)	Pathogenic (0.96)	+	+
7	–	–	–	–					−	−
8	–	–	–	–					−	−
9	APC	c.3949G > C	1317	15		Tolerated (0.19)	Benign (0.002)	Pathogenic (0.98)	+	+
		c.4360delA	1454	15	Novel	N/A	N/A	N/A	+	+
	KRAS	c.35G > C	12	2		Deleterious (0.02)	Possibly damaging(0.739)	Pathogenic (0.98)	+	+
	TP53	c.743G > A	248	7		Deleterious (0)	Probably damaging(0.992)	Pathogenic (0.98)	+	+
10	APC	c.4059_4060insT	1376	15	Novel	N/A	N/A	N/A	+	+
	TP53	c.845_846insCGGT	282	8	Novel	N/A	N/A	N/A	+	+
	PIK3CA	c.1633G > A	545	9		Deleterious (0.01)	Possibly damaging(0.868)	Pathogenic (0.97)	+	+
	FBXW7	c.1168dupT	390	7	Novel	N/A	N/A	N/A	+	+
11	KRAS	c.38G > A	13	2		Deleterious (0.03)	Possibly damaging(0.517)	Pathogenic (0.98)	+	+
	TP53	c.821T > A	274	8		Deleterious (0)	Probably damaging(0.994)	Pathogenic (1.0)	+	+
12	APC	c.4468_4474delCATTTTG	1490–92	15	Novel	N/A	N/A	N/A	+	+
	KRAS	c.35G > T	12	2		Deleterious (0)	Probably damaging(0.984)	Pathogenic (0.98)	+	+
	TP53	c.536A > G	179	5		Deleterious (0.01)	Probably damaging(0.971)	Pathogenic (0.99)	+	+
13	APC	c.3916G > T	1306	15		N/A	N/A	Pathogenic (0.96)	+	+
	TP53	c.524G > A	175	5		Tolerated (0.13)	Benign (0.209)	Pathogenic (0.99)	+	+
14	APC	c.4660_4661insA	1554	15		N/A	N/A	N/A	+	+
	KRAS	c.35G > A	12	2		Deleterious (0)	Benign (0.387)	Pathogenic (0.98)	+	+
	TP53	c.745A > G	249	7		Deleterious (0)	Probably damaging(0.994)	Neutral (0.41)	+	+
	PIK3CA	c.1624G > A	542	9		Tolerated (0.11)	Probably damaging(0.922)	Pathogenic (0.97)	+	+
15	APC	c.3957delT	1319	15		N/A	N/A	N/A	+	+
	KRAS	c.35G > A	12	2		Deleterious (0)	Benign (0.387)	Pathogenic (0.98)	+	+
	TP53	c.422G > A	141	5		Deleterious (0)	Probably damaging(1)	Pathogenic (0.99)	+	+
16	NRAS	c.181C > A	61	3		Deleterious (0.01)	Possibly damaging(0.751)	Pathogenic (0.99)	+	+
	TP53	c.761T > A	254	7		Deleterious (0)	Probably damaging(1)	Pathogenic (0.99)	+	+
17	APC	c.4455delT	1485	15		N/A	N/A	N/A	+	+
	KRAS	c.35G > C	12	2		Deleterious (0.02)	Possibly damaging(0.739)	Pathogenic (0.98)	+	+
	TP53	c.223_224dupCC	75	4	Novel	N/A	N/A	N/A	+	+
18	APC	c.4033G > T	1345	15		N/A	N/A	Pathogenic (0.85)	+	+
	TP53	c.524G > A	175	5		Tolerated (0.13)	Benign (0.209)	Pathogenic (0.99)	+	+
19	APC	c.4479G > A	1306	15		N/A	N/A	Neutral (0.46)	+	+
	TP53	c.406delC	136	5		N/A	N/A	N/A	+	+
20	TP53	c.638G > A	213	6		Deleterious (0)	Probably damaging(0.992)	Pathogenic (0.99)	+	+
		c.844C > T	282	8		Deleterious (0)	Probably damaging(0.999)	Pathogenic (0.99)	+	+
21	APC	c.3980C > G	1327	15		N/A	N/A	Pathogenic (0.90)	+	+
	TP53	c.437G > A	146	5		N/A	N/A	Neutral (0.17)	+	+
22	TP53	c.454_466delCCGCCCGGCACCC	152–156	5		N/A	N/A	N/A	+	+

A ‘ + ’ shows positive for mutation. ‘Wild’ signifies no mutation

QMC-PCR with HRM was used to validate identified mutations (Additional file 6: Fig. S1). The results proved that no false positives were generated.

In primary CRC cases, the median number of mutations per tumour was 2.63 (range = 0–7) over a median of 2.27 genes (range = 0–4). The matching metastases had a median of 2.54 mutations per tumour (range = 0–5) over a median of 2.27 genes (range = 0–4). The most commonly mutated genes in primary CRC cases include *TP53* (19 cases), *APC* (14 cases), and *KRAS* (8 cases) (see in Table 1). The metastatic cases had a similar common profile as follows: *TP53* (19 cases), *APC* (14 cases) and *KRAS* (8 cases). When the two tumour groups were combined, mutations were most frequently observed in *TP53* (86%), *APC* (44%), *KRAS* (36%), *PIK3CA* (9%), *SMAD4* (9%), *NRAS* (9%) and 4% for *FBXW7, BRAF, GNAS and CDH1*. However, for some cases multiple mutations were detected for a single gene. In total, 23 mutations were detected for *TP53*, *APC* (18), *KRAS* (10), *PIK3CA* (2), *SMAD4* (2), *NRAS* (2) and 1 mutation for *FBXW7, BRAF, GNAS and CDH1.*

### Mutations in matched pairs of primary and metastases CRCs

A total of 58 non-synonymous somatic variations in 10 genes were found in 22 primary tumours whereas 56 were found in metastatic cases. The mutant allele frequency was 1.03-fold higher in primary CRC than metastatic CRC. Four mutations in primary tumours were not seen in the metastatic tumours (private for primary) whilst two mutations present in the metastases were not seen in the primary tumours (private for metastasis). Although the paired primary and metastatic CRCs were not 100% identical, a Pearson’s correlation test shows no significant differences between them (p > 0.05).

Discrepancies between the primary and metastatic tumours were seen in four cases. As shown for case 1 in Table 1, the primary tumour was mutant for *KRAS* (G12V) and *TP53* (H179R) whereas the metastatic tumour was wild-type at these loci. This case also had mutations in *APC, SMAD4, KRAS* (Q61H) and *TP53* (V175A) which were identical in primary and metastasis. In case number 2, the primary tumour had an exon 8 *GNAS* mutation whereas the metastatic tumour was wild type. This case also had mutations for *APC*, *KRAS* and *SMAD4* which were identical in the primary and metastatic tumours. In case number 3, mutations were found for *NRAS* (Q61K) and *TP53* (I254N) in the metastatic tumour whereas the primary was wild-type. This case also had *APC* and *TP53* (G266E) mutations which were identical in the primary and metastasis. The primary tumour for case number 4 contained an exon 8 mutation in *CDH1* whereas in metastasis this gene was wild-type. *KRAS* and *TP53* mutations were identical this tumour pair.

### Tumour heterogeneity in primary versus metastatic CRC

**Fig. 1:**
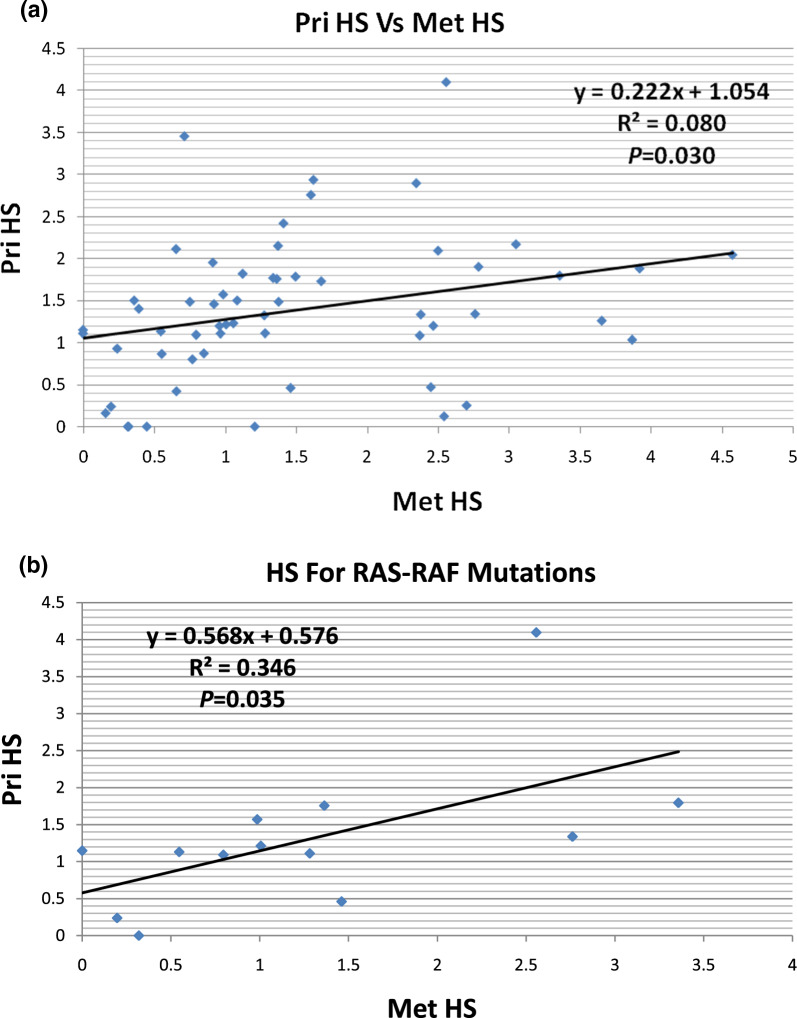
Pearson’s correlation of the heterogeneity score (HS) of primary (Pri) and metastatic (Met) CRC showing poor correlation of HS in **A** full somatic mutation profiles and **B** trunk drivers RAS-RAF (i.e. *KRAS, NRAS* and *BRAF*) mutation profiles

The comparison of the primary and metastatic tumours showed poor correlation in the heterogeneity scores for the somatic mutations (Pearson’s correlation coefficient, r = 0.284, r^2^ = 0.081, *P* = 0.030) (Fig. 1a). Furthermore, when the heterogeneity score data was analysed separately for trunk drivers (*APC, RAS-BRAF*), branching driver (*TP53*) and other (*PIK3CA, SMAD4, CDH1*, etc.) mutations, only the *RAS-BRAF* somatic mutations showed significant but poor correlation between primary and metastatic tumours (r = 0.588, r^2^ = 0.346, *P* = 0.035) (Fig. 1b). Heterogeneity score for somatic mutations in *APC, TP53,* and other genes did not show any correlation between primary and metastatic tumours (*APC*: r = 0.0122, r^2^ = 0.0001, *P* = 0.961; *TP53*: r = 0.256, r^2^ = 0.065, *P* = 0.264; other mutations: r = 0.383, r^2^ = 0.147, *P* = 0.395).

Moreover, we also compared the proportions of somatic mutations in the background of CNV, ATC, WT and STC between primary and metastatic tumours. Whilst the primary tumours showed the following indices: CNV = 35/58, STC = 19/58, ATC = 2/58 and WT = 2/58; the metastatic tumours showed CNV = 42/58, STC = 11/58, ATC = 1/58 and WT = 4/58. There was an inter-status change in heterogeneity states in 22/58 alleles comprising STC to CNV (STC/CNV) = 10/58, STC/WT = 3/58, CNV/STC = 4/58, CNV/ATC = 1/58, CNV/WT = 1/58, and ATC/CNV = 3/58. In addition, there was an intra-status change in the heterogeneity states in 19/58 alleles, comprising an increase in heterogeneity score in the CNV/CNV background in 8/58 alleles, a reduction in HS in CNV/CNV background in 10/58 alleles, and an increase in heterogeneity score in STC/STC background in 1/58 allele. No inter- or intra-status change in heterogeneity was seen in 17/58 (29.3%) alleles between primary and metastatic tumour pairs. In total, 41/58 mutant alleles (70.7%) showed discordance in the background heterogeneity states between primary and metastatic tumour pairs (z-score = 6.927, difference = 0.414, P < 0.0001, Additional file 5: Data_Somatic Mutation Profiles).

**Fig. 2:**
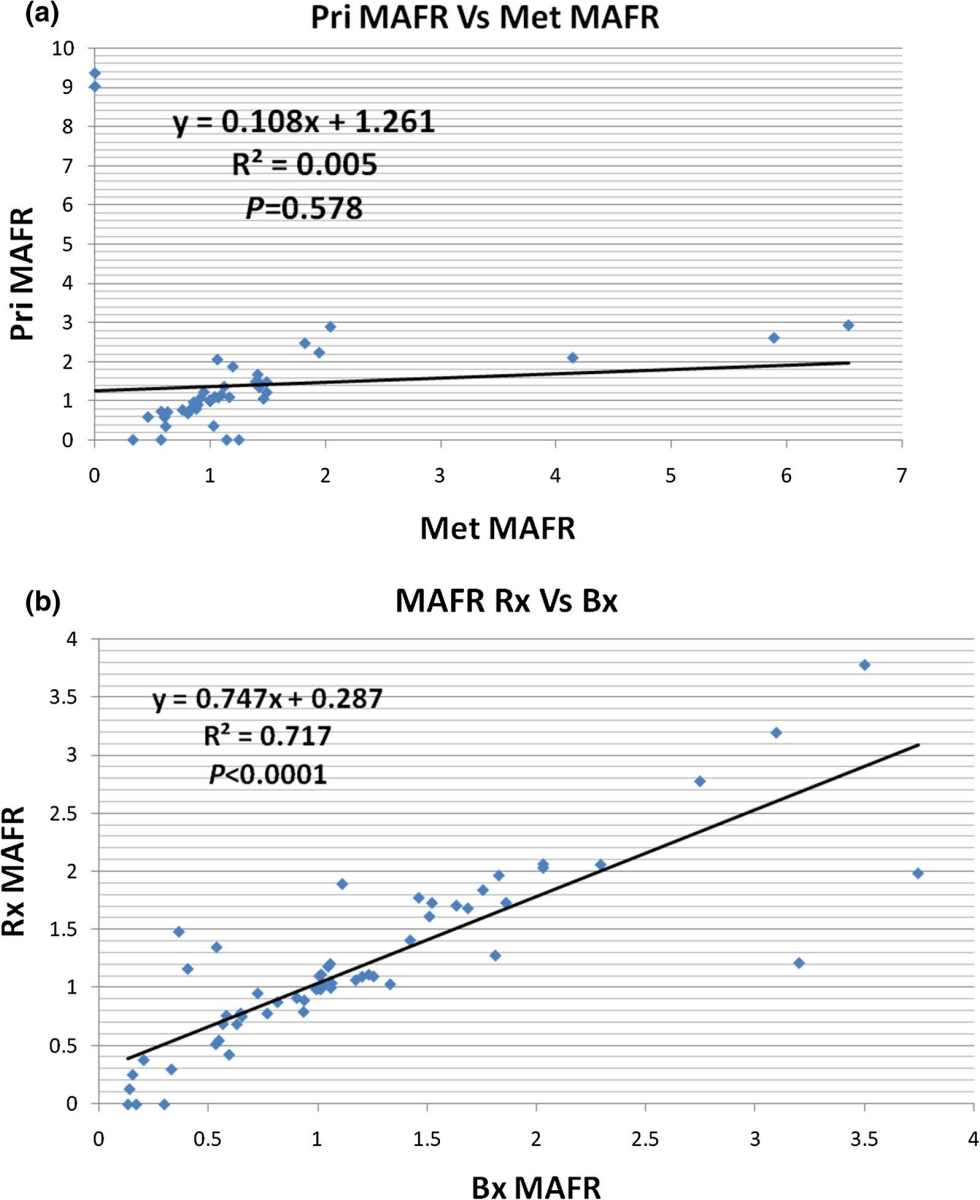
Pearson’s correlation of the mutant allele frequency ratios (MAFRs) of the full mutation profiles of primary and metastatic CRC showing **A** a lack of correlation between primary and metastatic cases MAFR, and **B** a strong correlation between resection (Rx) and biopsy (Bx) samples MAFR

Furthermore, the MAFR of primary tumours and their metastatic pairs were compared for any correlations. The results showed a lack of correlation in MAFR between primary and metastatic tumours (r = 0.074, r^2^ = 0.005, *P* = 0.578). In comparison the MAFR between resection samples (Rx) and their paired diagnostic biopsies (Bx) (see reference 26) showed a high correlation (r = 0.847, r^2^ = 0.717, *P* < 0.0001) (Fig. 2A &B).

### MiRNA quantification

#### miRNA profile

To identify aberrantly expressed miRNAs, the study quantified six miRNA levels in CRC. All assays were done in triplicates, and the Ct values of all targets were less than 25 in all the samples, ranging from 15.5 to 24.3, with SD less than 0.5 between replicate Ct values.The expression level of six miRNAs were analysed in 21/22 CRC pairs using RT-qPCR and the ΔΔCt method. The relative miRNA expressions of the six miRNAs in all 21 sample pairs were categorized into high, normal and low (Table 2, Additional file 3: miRNA).

**Table 2:** Concordance of miRNA expression between primary and metastatic tumours

	miRNA20a	miRNA21	miRNA29a	miRNA31	miRNA92	miRNA224
P1M1	LOW/LOW	LOW/LOW	HIGH/HIGH	**LOW/HIGH**	LOW/LOW	LOW/LOW
P2M2	HIGH/HIGH	LOW/LOW	HIGH/HIGH	HIGH/HIGH	**HIGH/LOW**	**HIGH/LOW**
P3M3	HIGH/HIGH	LOW/LOW	LOW/LOW	**LOW/NORMAL**	**HIGH/LOW**	LOW/LOW
P4M4	HIGH/HIGH	LOW/LOW	**LOW/NORMAL**	LOW/LOW	**LOW/HIGH**	LOW/LOW
P5M5	**LOW/HIGH**	LOW/LOW	LOW/LOW	LOW/LOW	LOW/LOW	**LOW/HIGH**
P6M6	LOW/LOW	LOW/LOW	LOW/LOW	LOW/LOW	LOW/LOW	LOW/LOW
P7M7	**LOW/HIGH**	LOW/LOW	LOW/LOW	LOW/LOW	LOW/LOW	**LOW/HIGH**
P8M8	**LOW/HIGH**	LOW/LOW	HIGH/HIGH	LOW/LOW	LOW/LOW	**LOW/HIGH**
P9M9	LOW/LOW	LOW/LOW	HIGH/HIGH	LOW/LOW	**HIGH/LOW**	LOW/LOW
P10M10	LOW/LOW	LOW/LOW	LOW/LOW	LOW/LOW	LOW/LOW	LOW/LOW
P11M11	LOW/LOW	LOW/LOW	HIGH/HIGH	**HIGH/LOW**	**HIGH/LOW**	LOW/LOW
P12M12	**HIGH/LOW**	LOW/LOW	**HIGH/LOW**	HIGH/HIGH	**HIGH/LOW**	HIGH/HIGH
P13M13	**HIGH/LOW**	LOW/LOW	HIGH/HIGH	LOW/LOW	**HIGH/LOW**	LOW/LOW
P14M14	HIGH/HIGH	LOW/LOW	LOW/LOW	LOW/LOW	HIGH/HIGH	LOW/LOW
P15M15	**HIGH/LOW**	LOW/LOW	LOW/LOW	LOW/LOW	LOW/LOW	LOW/LOW
P16M16	LOW/LOW	LOW/LOW	LOW/LOW	LOW/LOW	LOW/LOW	LOW/LOW
P17M17	**HIGH/LOW**	**LOW/HIGH**	**LOW/HIGH**	LOW/LOW	HIGH/HIGH	LOW/LOW
P18M18	HIGH/HIGH	LOW/LOW	**HIGH/LOW**	LOW/LOW	**HIGH/LOW**	LOW/LOW
P19M19	HIGH/HIGH	LOW/LOW	**HIGH/LOW**	LOW/LOW	**HIGH/LOW**	LOW/LOW
P20M20	**HIGH/LOW**	LOW/LOW	**HIGH/LOW**	LOW/LOW	**HIGH/LOW**	LOW/LOW
P22M22	**NORMAL/LOW**	LOW/LOW	**HIGH/LOW**	**LOW/HIGH**	**HIGH/LOW**	LOW/LOW

Tables in bold fonts represent altered relative expression of miRNA between primary and matching metastatic tumours

The primary versus metastatic tumour concordance for all six miRNAs were calculated as follows: miRNA20 (Kappa = 0.185, SE of Kappa = 0.194, 95% confidence interval [CI] = − 0.201 to 0.572); miRNA29a (Kappa = 0.382, SE = 0.169, 95% CI = 0.050 to 0.714); miRNA31 (Kappa = 0.400, SE = 0.239, 95% CI = − 0.069 to 0.868); miRNA92 (Kappa = 0.049, SE = 0.134, 95% CI = − 0.213 to 0.312) and miRNA224 (Kappa = 0.236, SE = 0.262, 95% CI = − 0.278 to 0.751) (see Table 2). Whilst miRNA20a expression only showed fair agreement between primary and metastatic tumours, miRNA29a, miRNA31, miRNA92 and miRNA224 expression showed no significant concordance between the two tumour groups. Kappa statistics could not be computed for miRNA21 because 20/21 (95.24%) of the sample pairs clustered in the low/low concordance group and therefore zero sample pairs were present in seven of the nine groups used in computing the statistics.

Furthermore, we investigated the differential expression of miRNAs in metastatic tumours relative to their primary tumour counterparts using tumour cellularity-normalised relative expression ratios (PM-RE) (see Table 3, Additional file 3: Data_miRNA). Four of the six miRNAs showed significant changes in expression in the metastatic tumours relative to the primary CRCas follows: miRNA20a (altered PM-RE = 12/21 tumour pairs, z-score = 1.323, P = 0.1859, adjusted P = 0.1859), miRNA21 (altered PM-RE = 12/21 pairs, z-score = 1.323, P = 0.1859, adjusted P = 0.1859), miRNA29a (altered PM-RE = 13/21 pairs, z-score = 2.247, P = 0.0247, adjusted P = 0.03705), miRNA31(altered PM-RE = 17/21 pairs, z-score = 7.224, P < 0.0001, adjusted P = 0.0002), miRNA92 (altered PM-RE = 16/21 pairs, z-score = 5.636, P < 0.0001, adjusted P = 0.0002) and miRNA224 (altered PM-RE = 15/21 pairs, z-score = 4.347, P < 0.0001, adjusted P = 0.0002). Furthermore, 57.14–80.95% of the primary-metastases tumour pairs showed altered P-M relative expression in all (100%) of the miRNAs tested. No consistent pattern of alteration was observed for any of the miRNA tested.

**Table 3:** Relative Expression of miRNAs between primary and metastatic tumours

	miRNA20a	miRNA21	miRNA29a	miRNA31	miRNA92	miRNA224
P1M1	**0.03585778**	**0.380851302**	1.06875214	**0.016550802**	1.376933695	**0.348727511**
P2M2	1.09645	0.744853227	0.634025639	**0.28162107**	**43.60894807**	**19.04037414**
P3M3	1.692489273	**0.19090725**	**0.29605899**	**0.066645422**	**16.1857784**	**0.117350079**
P4M4	1.827198884	**0.415489365**	**0.385843106**	**0.125200289**	**0.089000864**	**0.038028682**
P5M5	**0.165149779**	**0.405168741**	1.017076664	**0.339575209**	**0.423256334**	**0.035356511**
P6M6	**9.032603896**	1.486654886	**2.40478338**	**0.446682873**	1.377598043	**0.346092903**
P7M7	**0.086177313**	**5.109981798**	**2.836372188**	**0.223606276**	0.545315661	**0.025755563**
P8M8	**0.036665352**	0.595136416	0.609622383	0.672004739	**15.27884066**	**0.050267673**
P9M9	0.791830378	**0.433974719**	**7.749368098**	1.218222488	**39.81362463**	**2.231345879**
P10M10	1.394741901	**0.453943997**	**0.435070972**	**0.40896525**	**0.073191164**	**0.06857601**
P11M11	1.114735233	1.975651409	1.682212004	**42.32879328**	**153.2597858**	0.831435329
P12M12	**14.54973359**	**3.999988297**	**32.62794428**	**0.375873656**	**18.4565922**	1.431082595
P13M13	**14.40626287**	0.631038396	1.104248944	**0.291989528**	**18.90293824**	1.304937537
P14M14	0.928942468	1.719170222	1.31107852	**6.881585216**	1.284949901	**0.353125807**
P15M15	**20.67402285**	1.515992607	1.799639358	1.184994819	1.523399321	**0.250151106**
P16M16	1.228038849	0.905169764	**3.674614842**	**0.411291757**	**34.59836117**	0.896321213
P17M17	**26.635528**	**0.026303388**	**0.052693645**	**0.041371318**	**0.138602656**	**0.035727369**
P18M18	0.67089814	**2.075354337**	**18.2393941**	**5.40911457**	**17.46464893**	1.408999347
P19M19	**0.114868611**	**11.72405865**	**212.6193329**	**0.330265922**	**4589.949761**	**15.08672611**
P20M20	**11.96089471**	1.273562362	**10.3128689**	0.562961827	**25.40671426**	1.975487901
P22M22	**10.67585162**	**3.782676577**	**20.47102061**	**0.034650289**	**87.78290945**	**3.899262033**

Tables in bold fonts represent altered relative expression of miRNA between primary and matching metastatic tumours

### Cut-off point detection of miRNAs

Before running tumour samples to detect expression levels of miRNAs, we sought to find the cut-off point to show high or low expression. To achieve this goal, we extracted RNA from 20 pure normal colon tissue that were pooled with an equal amount from each sample. The expression level of all miRNAs was measured in all normal colon tissue samples after comparing with the pooled sample. On average the minimum fold of expression of all miRNAs in normal colon tissue was 0.5 and the highest was 1.5 (using < 0.5 fold as showing downregulation and > 1.5 fold as showing up-regulation). 

### Reverse phase protein assay RPPA

Whole protein lysates from 20/22 primary CRCs and their matched metastatic tumours were obtained and analysedfor the expression of 22 different proteins by reverse phase protein array. The samples were run in triplicates and the mean of these replicates for each target in each sample was calculated (Additional file 7: Fig. S2).

#### RPPA analysis methodology validation

First, the validity of the differential biomarker expression method we applied was tested using concordance in alteration status (upregulated, normal, downregulated) between proteins and their isoforms/modified forms and between proteins that are closely related functionally (see Additional file 4: Data_RPPA Markers). The following results were obtained: CD34/CD31(Kappa = 0.696, SE = 0.135, 95% C.I. = 0.432 to 0.960; substantial agreement); P85/P110 (Kappa = 0.005, SE = 0.183, 95% C.I. = – 0.354 to 0.363; no agreement); PTEN/phosphoPTEN (Kappa = 0.681, SE = 0.144, 95% C.I. = 0.400 to 0.963; substantial agreement); RAS/RASA1(Kappa = 0.897, SE = 0.101, 95% C.I. = 0.699 to 1.000; almost perfect agreement); phosphoAKT-serine/phosphoAKT-threonine (Kappa = 0.921, SE = 0.078, 95% C.I. = 0.768 to 1.000; almost perfect agreement); mtor/phosphoAKT-serine (Kappa = 0.762, SE = 0.129, 95% C.I. = 0.508 to 1.000; substantial agreement); mtor/phosphoAKT-threonine (Kappa = 0.843, SE = 0.107, 95% C.I. = 0.632 to 1.000; almost perfect agreement); TGFBRII/SMAD4 (Kappa = 0.835, SE = 0.113, 95% C.I. = 0.613 to 1.000, almost perfect agreement). The results showed that 7 out of the 8 pairs of markers tested had at least substantial agreement in alteration status using our differential biomarker expression approach, thereby confirming the validity of our method.

#### Protein pathways analyses

Analysis of the protein expression in a *biological context* revealed that all the pathways examined had significant alterations in the tumour-pair population using our 50% criterion. Whilst for the CAM-EMT pathway 14/20 tumour pairs showed alteration in status, for the RAS-RAF-MEK, PI3K-AKT-PTEN, TGFBRII/SMAD4, apoptosis and transcriptional mis-regulation pathways,15/20, 13/20, 11/20, 13/20, and 16/20 tumour pairs, respectively, showed alteration in relative expression status (see Additional file 1: Data_Biomarker Pathways). Furthermore, 16 of 20 (80%) tumour pairs showed alteration in at least 3/6 (50%) of the pathways analysed, evidence that the overall protein marker expression profiles of primary and metastatic tumours were significantly different.

## Discussion

The overall five-year survival for CRC is 57% and metastasis will occur in up to 50% of all patients. Metastasis is responsible for most cancer deaths even with improved surgery and chemotherapy [28]. Elucidation of the cellular and molecular mechanisms of metastasis may facilitatethe future development of diagnostic and therapeutic interventions and foster improve patient prognostication. New trends in cancer therapies are increasingly moving towards personalised medicine which targets the molecular alterations that cause cancer progression. With these strategies coming into play, the genotyping of patients with advanced CRC is now being performed as a part of standard clinical practice. In particular, treatment with anti-EGFR therapy requires the determination of *KRAS* mutation status in patients with metastatic CRC [29]. However, it remains unknown whether the molecular milieux of primary lesions are identical to those of their matching metastatic tumours and if performing molecular testing in primary tumours alone is sufficient for the targeted treatment of metastatic tumours.

The National Comprehensive Cancer Network (NCCN) recommends testing either the primary tumour or the metastatic lesion based on the results ofseveral studies that highlighted high concordance (> 95%) of *KRAS* mutations between primary CRCs and their matched metastases [30, 31]. Similar to the studies on which the NCCN based its recommendations, our study found a high concordance in somatic mutation profiles between primary and metastatic tumours. In contrast, however, are the reports from some other studies which found significant discordance in mutation profiles of primary tumours and their matched metastases [32, 33].

However, it must be noted that patients’ prognoses and therapeutic responses depend on other factors apart from the presence of one somatic mutation type or the other. For example, intra-tumour heterogeneity—both spatial and temporal—is emerging as a relevant factor in therapy response and prognosis [23–25, 33–36]. Heterogeneity can be measured in terms of the background status of individual somatic mutation types [23–25]. That is, a specific mutation type can exist in only some tumour cells, in all tumour cells or in a background of copy number variation. It has been suggested that tumour heterogeneity may account for therapy resistance in the presence of matched targeted treatments and somatic mutations [35]. Tumour heterogeneity may partly explain the reason why some tumours with wild-type *KRAS* status respond to anti-EGFR therapy while others do not [37–39]. To put it in perspective, response to anti-EGFR therapy can differ between two patients with the same *KRAS* mutation status if such mutations were present in only some tumour cells in one patient, but occur in all the tumour cells, or in a copy number variation background, in the other patient. Likewise, if the background status of predictive markers differs between primary and metastatic tumours the response to therapy in the cells in the primary site can differ than those in the metastatic one [37–39]. In this study we showed significant discordance in tumour heterogeneity statuses between primary tumours and their matched metastases.

Furthermore, recent studies have demonstrated that the mutant allele frequency (MAF) *changes* in the metastatic tumour are important in therapy response [35]. Many investigators have shown that tumour sub-clones with minor frequency in the primary tumour can become prominent, therapy-resistant clones in the metastatic disease [40–42]. *Differential* mutant allele frequencies can therefore indicate differential prognosis and response to therapy. Therefore, *actual* mutant allele frequencies may be more informative than the currently adopted present-or-absent mutation approach, and may find more clinical importance in the near future. However, any method of MAF assessment must take into cognisance the diluting effect of ‘contaminating’ stromal DNA and tumour cellularity on the measurement of MAF. In this study we used the mutant allele frequency *ratios* (MAFRs) to compare the MAFs between primary and metastatic tumours, as previously explained [26], and found a poor or weak correlation between the two tumour groups. The use of MAFR to compare the MAF between primary and metastatic tumours eliminates the confounding effects of differences in tumour cellularity between the primary tumour and its matched metastasis.

MicroRNAs are small single-stranded RNAs of approximately 22 nucleotides in length. They are involved in gene regulation and function by down-regulating gene expression via the inhibition of mRNA expression or via the promotion of target RNA degradation [43]. Through their gene regulatory actions, they play important roles in many biological processes under physiological or pathological conditions, including during development [44], cell metabolism [45], cell proliferation, migration, apoptosis, cell differentiation [43], immune response [46], tumourigenesis, and metastasis [47, 48]. In tumourigenesis, miRNAs can function as either oncogenes or tumour suppressors depending on the mRNA targets expressed by the tumour cells [43]. Furthermore, miRNA have been shown to function as metastasis promoters or inhibitors through the regulation of invasion, migration, colonization, cancer stem cell properties, EMT and the microenvironment [43, 48, 49]. For example, the miR-17-92 cluster which includes miRNA20a and miRNA92a have both anti-apoptotic and pro-apoptotic, as well as proliferative and anti-proliferative functions in tumourigenesis, depending on the cellular context [50]. MiRNA21 is up-regulated in various cancers and targets pdcd4 to promote invasion, intravasation and metastasis [51–54]. In addition, it is associated with poor survival, advanced tumour stage and poor therapeutic outcome [55]. MiRNA29a promotes colorectal cancer cell metastasis via the down-regulation of KLF4 and e-cadherin, and up-regulation of MMP2 [53, 54]. It also suppresses colon cancer cell growth via the inhibition of the PTEN/Akt/GSK3β and Wnt/β-catenin signalling pathways [56]. Additionally, it is associated with longer disease-free survival in stage II colon cancer [57] MiRNA31 promotes colon cancer cell proliferation, invasion and migration through the suppression of SATB2; and the expression of miRNA31 in clinical tumours is associated with the more aggressive and poor prognostic phenotypes of CRC [58]. MiRNA224 activates the Wnt/β-catenin signalling, down-regulates GSK3β, and promotes aggressive phenotype of colorectal cancer cells [54, 59]. Of importance to cancer therapy, miRNAs of different types have been shown to regulate the expression of cytochrome P450 and other drug metabolizing genes [60, 61]. Of more clinical importance, perhaps, are the findings that suggest that miRNAs are potential biomarkers of response to common cancer therapeutics such as chemotherapeutic drugs (5-fluorouracil [62, 63], S-1 [63], paclitaxel [64], cisplatin [64, 65], gemcitabine [66]), hormonal therapy (tamoxifen [67], glucocorticoids [68]), tyrosine kinase inhibitors (sorafenib [69], anti-EGFR TKI [70]), and radiotherapy [71]).

During tumour progression gene expression patterns change and this is consequent upon changes in gene regulatory patterns [72]. Without these changes in gene regulation and gene expression patterns, primary tumours would not metastasize. We measured the expression of miRNAs in primary and metastatic tumour pairs for concordance of expression, as well as expression of miRNA in metastatic tumours relative to primary tumours. We found loss of concordance in miRNA expression and significant alterationsin miRNA relative expression between primary and matched metastatic tumours. Our findings are in conformity with previous reports from miRNA studies. For example, Ellermeier et al. [73] interrogated 377 miRNAs in 19 CRC primaries and their matched metastases and found that 16/377 miRNA had differential miRNA expression between primary colorectal tumours and their matched liver metastases. In that studyit was found that whilst some miRNAs were down-regulated in the metastatic tumours, others were up-regulated, which is in keeping with our findings. Furthermore, Feiersinger et al. [51] found that miRNA21 expression was significantly reduced in liver metastases compared to primary colorectal tumours in a study comparing miRNA expression in 29 CRC patients. In addition, Pizzini et al. [74] found that miRNA146a and miRNA201 were significantly altered in metastatic colorectal tumours relative to their matched primaries. Moreover, Vychytilova-Faltejskova et al. [75], using genome-wide expression profiling, screened 86 primary CRC and their matched metastases for quantification of 752 human miRNAs and six endogenous controls, and found that 33 miRNAs have significantly deregulated expression in metastatic tissue. These miRNAs included miR-122, miR-122*, and miR-885-5p which were significantly higher in metastatic tissue compared to primary tumours, and miR-143 miR-10b, and miR-28-5p which were significantly reduced in metastatic tumours in the liver. However, in contrast to these studies, we did not observe any consistent pattern of alteration between primary and metastatic CRC for any of the miRNAs tested.

Although the miRNA profiles have been used—at least at clinical research level—in the prognostication of CRC patients [55, 57–59], the prognostic values of combined primary-metastasis miRNA profiles are currently unknown. The miRNA profiling of the metastatic tumour may provide an opportunity for the improved prognostication of CRC patients. Our findings—and those of others mentioned above –also have profound implications for the treatment of CRC patients. As miRNA expression has been associated with differential response to drug therapy [60–70], differential miRNA expression between primary tumour and their matched metastasis would imply that drug treatment given for a primary tumour can be ineffective for treating metastatic deposits in the same patient.

To analyse our RPPA data, we developed a novel approach which utilizes the standard deviation as the cut-off for adjudging changes in expression levels between primary tumours and their matched metastases, and validated the approach by comparing the alteration patterns found for proteins and their isoforms, as well as for proteins which are functionally related. We reasoned that if our approach was valid, functionally related proteins and protein isoforms should show similar alteration patterns. Having validated our approach, we proceeded to analyse the protein biomarker expression data using a stringent biological pathway analysis approach. We used the alteration of 50% of the pathways analysed as the cut-off for significant alteration of overall expression profile between primary tumours and their matched metastases, since alteration in any one pathway in biological systems is associated with perturbation of (an)other pathway(s) [76]. We found that 80% of the primary-metastasis pairs exhibited alterations in at least 3 of the 6 pathways analysed, suggesting that the expression profiles of primary tumours are different from those of their matched metastases. The protein biomarkers tested in this study belong to established oncogenic, tumour suppressor and apoptotic pathways [27, 76]. Our findings are in keeping with the established notion that the metastasis of primary tumour cells is associated with or dictated by changes or alterations in gene expression patterns of the cells [72]. However, we did not find any consistent pattern of alteration between primary and metastatic CRC for any of the protein pathways tested. But more importantly, the proteins analysed in this study are markers of drug responsiveness [77]. For example, whilst mtor and PI3K expression are biomarkers of Dactolisib response in solid tumours [78], PTEN is a marker for trastuzumab, cetuximab, gefitinib and erlotinib response [77, 79–81]. Therefore, the loss of biomarker in either a primary tumour or its matched metastases would lead to partial drug response or even treatment failure in any individual patient.

Differential expression of protein and miRNA markers between primary tumours and their matched metastases is evidence of temporal heterogeneity of CRC [23–25, 34–36]. And, has been mentioned for miRNA expression, a combined primary-metastasis protein expression profiling would give a more complete molecular portrait of a patient’s disease. than profiling either the primary or metastatic tumour alone. Ellemeier et al. [73] suggested that it may be beneficial for improved prognosis—and therapeutic response, we might add—to always probe the differences in the molecular alterations in both primary and metastatic tumours for each patient, rather than test either the primary or metastatic tumour. The presence of differential molecular alterations between matched primary-metastasis tumour pairs throws up opportunities—rather than challenges—for improved personalisation of drug regimens and prognostication of the disease.

In summary, we have evaluated primary CRCs and their matched metastases at the genomic, transcriptomic and proteomic levels, and found that primary tumours are inherently different from their corresponding metastases. These findings have both prognostic and therapeutic implications for CRC patients.

### Supplementary Information

**Additional file 1.** Data_biomarker pathways.

**Additional file 2.** Data_general.

**Additional file 3.** Data_miRNA.

**Additional file 4. **Data_RPPA markers.

**Additional file 5.** Data_somatic mutation profile.

**Additional file 6. Fig. S1.** High Resolution Melting Difference Curve showing aberrant melting at APC exon 15 for samples 3, 5, 8, 9 and 12.

**Additional file 7. Fig. S2.** Heat-map showing different signalling pathway intermediates studied in primary CRC (22) and metastasis (22) using RPPA. Rows represent the different signalling molecules studied. Green and red denote markers that are present at low levels

**Additional file 8.** NGS.

## Data Availability

All datasets on which the conclusions of this paper rely have been presented in the main manuscript and in the additional supporting files. Additionally, raw data can be accessed on request.
