# Supplementary figures and images for: Investigating genomic, proteomic, and post-transcriptional regulation profiles in colorectal cancer: a comparative study between primary tumors and associated metastases
Source: Cancer Cell Int. 2023 Sep 5;23:192. doi: 10.1186/s12935-023-03020-7 (PMC10478430; doi:10.1186/s12935-023-03020-7)

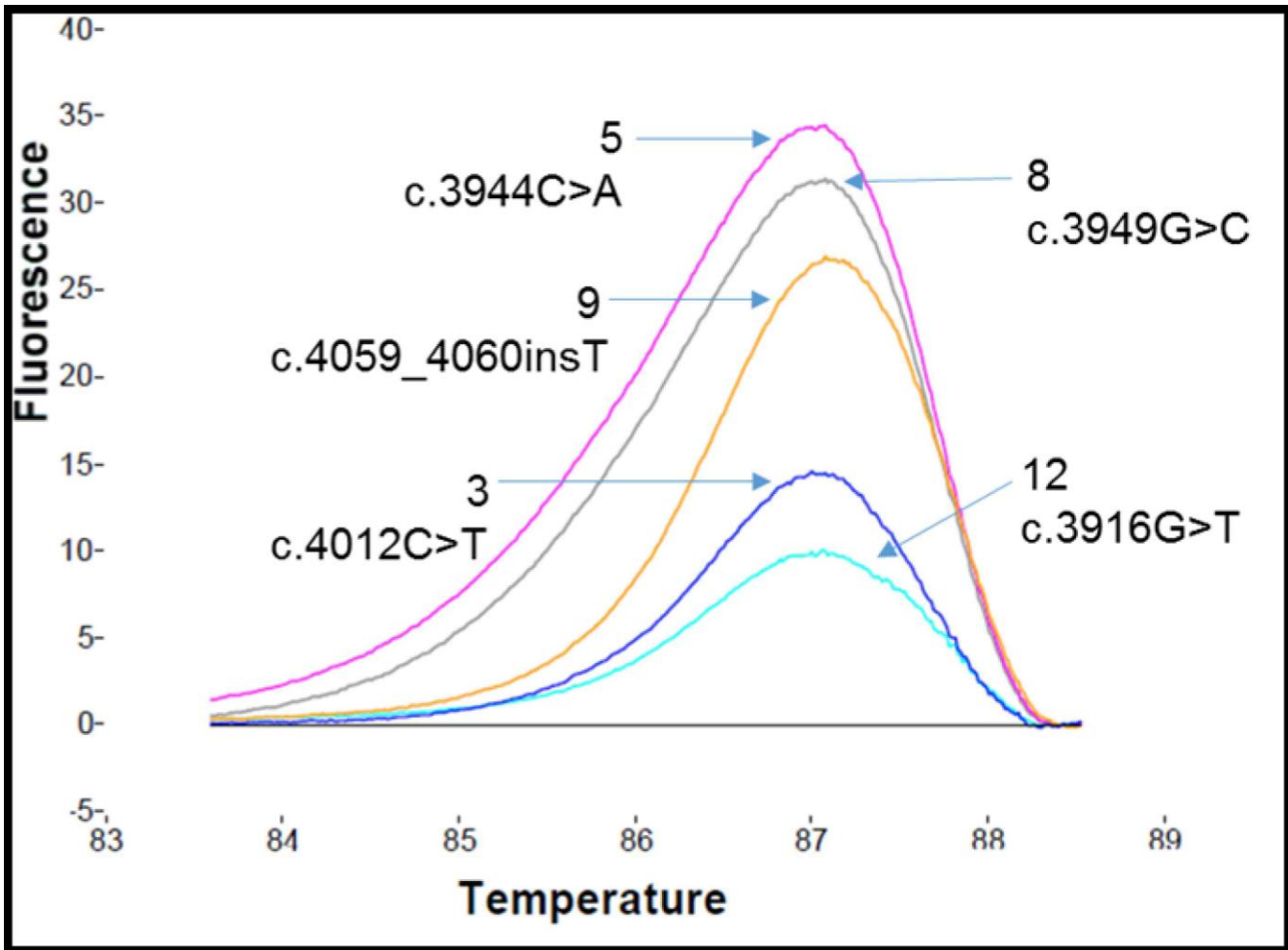

Supplement: Supplementary file 6 — Additional file 6. Fig. S1. High Resolution Melting Difference Curve showing aberrant melting at APC exon 15 for samples 3, 5, 8, 9 and 12. [file 12935_2023_3020_MOESM6_ESM.pdf]
