# Supplementary material for: Investigating genomic, proteomic, and post-transcriptional regulation profiles in colorectal cancer: a comparative study between primary tumors and associated metastases
Source: Cancer Cell Int. 2023 Sep 5;23:192. doi: 10.1186/s12935-023-03020-7 (PMC10478430; doi:10.1186/s12935-023-03020-7)
Supplement: Supplementary file 7 — Additional file 7. Fig. S2. Heat-map showing different signalling pathway intermediates studied in primary CRC (22) and metastasis (22) using RPPA. Rows represent the different signalling molecules studied. Green and red denote markers that are present at low levels [file 12935_2023_3020_MOESM7_ESM.pdf]

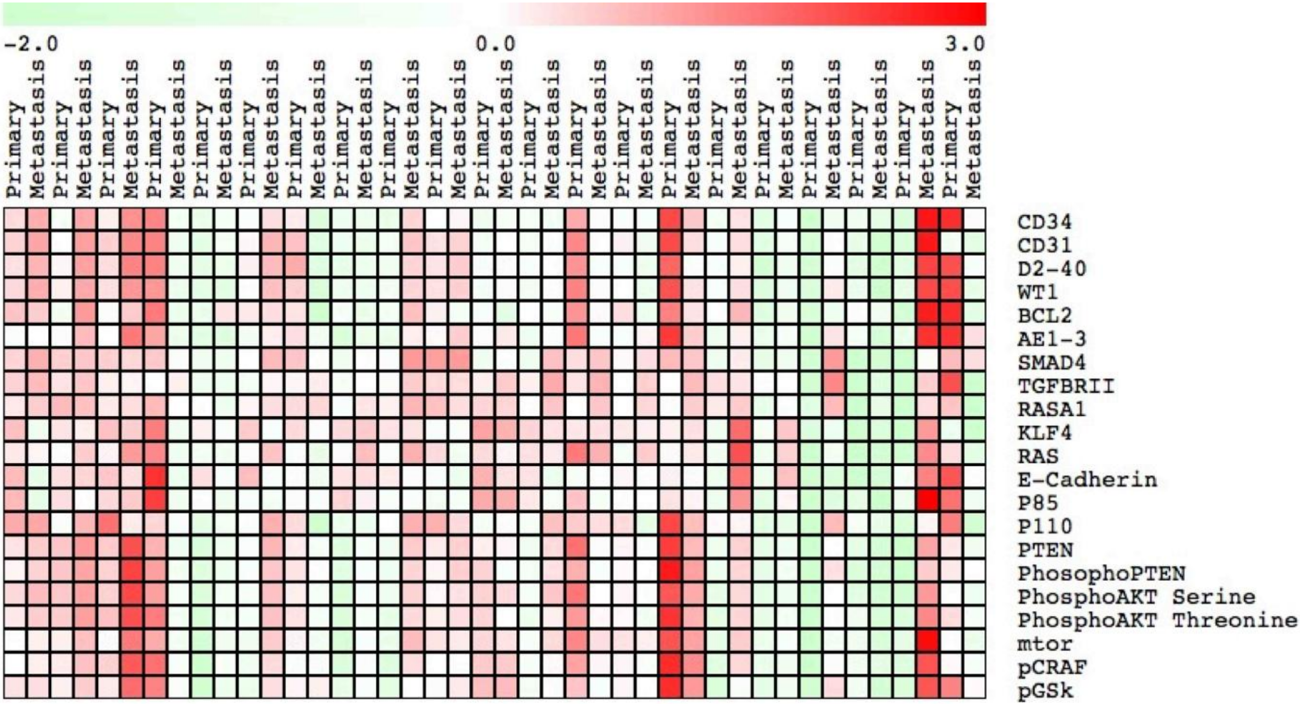
-2.0
0.0
3.0
Primary
Metastasis
Primary
Metastasis
Primary
Metastasis
Primary
Metastasis
Primary
Metastasis
Primary
Metastasis
Primary
Metastasis
Primary
Metastasis
Primary
Metastasis
Primary
Metastasis
Primary
Metastasis
Primary
Metastasis
Primary
Metastasis
Primary
Metastasis
Primary
Metastasis
Primary
Metastasis
Primary
Metastasis
Primary
Metastasis
Primary
Metastasis
Primary
Metastasis
Primary
Metastasis
CD34
CD31
D2-40
WT1
BCL2
AE1-3
SMAD4
TGFBRII
RASA1
KLF4
RAS
E-Cadherin
P85
P110
PTEN
PhosophoPTEN
PhosphoAKT Serine
PhosphoAKT Threonine
mtor
pCRAF
pGSk
