# Supplementary material for: Investigating genomic, proteomic, and post-transcriptional regulation profiles in colorectal cancer: a comparative study between primary tumors and associated metastases
Source: Cancer Cell Int. 2023 Sep 5;23:192. doi: 10.1186/s12935-023-03020-7 (PMC10478430; doi:10.1186/s12935-023-03020-7)
Supplement: Supplementary file 8 — Additional file 8. NGS. [file 12935_2023_3020_MOESM8_ESM.docx]

**Next Generation Sequencing**

Mutation profiles were determined using the TruSight tumour kit and samples were run on an Illumina MiSeq DNA analyzer The TruSight tumour kit offers deep coverage of 26 genes across 175 amplicons of relevant content from CAP (College of American Pathologists) and NCCN (The National Comprehensive Cancer Network^®^) guidelines (a minimum 1000X coverage and an average of 7000X coverage). The genes included in this kit were carefully selected by Illumina as being involved in solid tumours including lung, colon, melanoma, gastric and ovarian carcinomas and provide coverage of all exons in tumour suppressor genes and exon coding regions where variation has been catalogued in the COSMIC database (Illumina, California, USA).

Each sample underwent a quality control step to test for template integrity in accordance with the manufacturer’s instructions. PCR based library preparation was carried out in accordance with the manufacturers’ instructions. The libraries consisted of PCR products of the targeted sequences flanked by common adapters (required for cluster generation and sequencing) and index sequences used to identify individual samples. The libraries for each sample were cleaned up and diluted to a final concentration of 4nM. Following this, all libraries were pooled into a single tube. Captured libraries were amplified and sequenced as paired-end reads on a MiSeq flow cell. A total of 12 samples were run on each flow cell.

**NGS Data analysis**

After sequencing, the raw signal data was analysed using MiSeq Reporter v2.1 software.. After primary data analysis, detected sequence variants (including single nucleotide variants (SNVs) and insertions or deletions (indels)) were assembled in a variant call file format generated by the MiSeq Reporter Program. Variant filtering and annotation was performed with Variantstudio^TM^ v2.1analyser. Single nucleotide variants (SNVs) or insertions/deletions (indels) were defined based on the following criteria: (1) were present in both pools (i.e. were present in both forward and reverse sequencing), (2) read depth of 1000x or an average depth of 500x per pool, (3) have >3% variant frequency in the merged variant call (VCF) files, (4) have a minimum Q-score of 20 (phredQ score: Q-score is a prediction of the possibility of an incorrect base call. A higher Q-score means that a base call is less likely to be incorrect and more reliable), (5) must not be a polymorphism according to dbSNP (Illumina, USA). For the evaluation of mutation artefacts, criterion (1) and (3) were excluded. For the analysis of the limit of detection, criterion (3) was excluded.
